# LC-3和P62在非小细胞肺癌中的表达及其临床特征分析

**DOI:** 10.3779/j.issn.1009-3419.2018.06.04

**Published:** 2018-06-20

**Authors:** 璁 王, 永文 李, 颖 李, 颢 宫, 洪兵 张, 茵 袁, 伟婷 李, 红雨 刘, 军 陈

**Affiliations:** 1 300052 天津，天津医科大学总医院肺部肿瘤外科 Department of Lung Cancer Surgery, Tianjin 300052, China; 2 300052 天津，天津市肺癌研究所，天津市肺癌转移与肿瘤微环境实验室 Tianjin Key Laboratory of Lung Cancer Metastasis and Tumor Microenvironment, Tianjin Lung Cancer Institute, Tianjin Medical University General Hospital, Tianjin 300052, China

**Keywords:** LC-3, P62, 自噬, 非小细胞肺癌, 免疫组织化学, LC-3, P62, Autophagy, Non-small cell lung cancer, Immunohistochemistry

## Abstract

**背景与目的:**

LC-3和P62是参与自噬过程的两个重要蛋白，在许多恶性肿瘤中高表达并与预后有关。本研究的目的在于探讨自噬标记物LC-3、P62蛋白在非小细胞肺癌中的表达及其临床特征。

**方法:**

应用免疫组化法检测66例非小细胞肺癌患者肿瘤组织中LC-3和P62的表达情况，分析其表达状态与临床特征的关系。

**结果:**

LC-3在非小细胞肺癌组织中阳性表达率为40.9%（27/66），P62在非小细胞肺癌组织中阳性表达率为65.2%（43/66）。LC-3的阳性表达与患者的病理类型及转移情况明显相关（*P* < 0.05）；而P62的阳性表达与患者的病理类型，临床分期及淋巴结转移明显相关（*P* < 0.05）。LC-3和P62的阳性表达与患者的性别、年龄和吸烟史无明显相关性（*P* > 0.05）。LC-3与P62在非小细胞肺癌组织中的表达呈负相关性（r_s_=-0.065, *P* < 0.001）。*Kaplan-Meier*分析显示LC-3蛋白阳性表达的患者5年生存率明显高于阴性表达的患者（*P* < 0.05），而P62蛋白的阳性表达确与患者的生存率无显著相关性。

**结论:**

LC-3和P62在非小细胞肺癌组织中呈现异常表达，提示自噬参与了非小细胞肺癌的发生及发展过程。

## Abstract (en)

**Background and objective:**

LC-3 and P62, two of important autophagy-related proteins, were reported highly expressed in many kinds of human malignancies and associated with outcomes of the patients. The purpose of this study was to investigate the expression status of LC-3 and P62 in non-small cell lung cancer patients and define the clinical-pathologic features.

**Methods:**

66 cases of non-small cell lung cancer patients were employed. The expression of LC-3 and P62 were detected by immunohistochemistry.

**Results:**

LC-3 was positive stained in 27 out of 66 cases (40.9%) and P62 was positive stained in 43 out of 66 cases (65.2%). LC-3 positive staining was more frequently in squamous cell carcinoma patients (*P* < 0.05); while P62 positive staining was more frequently in late-stage adenocarcinoma patients with metastasis (*P* < 0.05). There was a significant negative correlation between LC-3 and P62 expressions in non-small cell lung cancer tissues (r_s_=-0.065, *P* < 0.001). *Kaplan-Meier* analysis showed that patients with positive LC-3 expression had favorable clinical outcomes compared with the patients with negative LC-3 expression (*P* < 0.05).

**Conclusion:**

LC-3 and P62 showed abnormal expression in non-small cell lung cancer tissues, suggesting that autophagy is involved in the occurrence and development of NSCLC.

肺癌是目前最常见的恶性肿瘤，其发病率与死亡率在全球居各种肿瘤之首，虽然手术、化疗、分子靶向治疗等治疗手段取得了巨大进步，但其5年生存率仍徘徊在15%左右^[1]^，其中，非小细胞肺癌（non-small cell lung cancer, NSCLC）占肺癌总量的80%-85%。由于肺癌发病隐匿，30%-50%的患者在确诊时已处于疾病的中晚期，预后极差，5年生存率小于10%。因此，明确肺癌的发病机理必将对肺癌的诊治起到至关重要的作用。自噬（autophagy）是一个进化保守的细胞内分解代谢过程，细胞利用溶酶体降解自身受损的细胞器和大分子物质，是真核细胞特有的生命现象。现有的证据表明自噬的异常与肿瘤的发生发展关系密切^[2]^，但是其作用机制还不明。现有的观点认为，在肿瘤的早期，自噬可降解有害的蛋白，维持内环境的稳定，抑制肿瘤的发展; 而在肿瘤的晚期，自噬可促进肿瘤的发展和转移。因此，研究自噬在肺癌发生发展中的作用是十分必要的。

微管相关蛋白轻链3（microtubule associated protein 1 light chain 3, LC3）是哺乳动物细胞中酵母ATG8（Ant7/Apg8）基因的同源物，是自噬的一种标记性基因，其表达水平与自噬活性密切相关，是目前公认的自噬标记基因^[3, 4]^。P62是由原癌基因*c-myc*编码的基因表达产物，它可以与泛素化的蛋白质相结合，将其转运至自噬小体内进行降解，因此它是反映自噬活性的标记蛋白。目前，有关LC3和P62在NSCLC中表达情况的研究较少^[5-7]^。本研究通过免疫组化法联合检测LC-3、P62在NSCLC组织中的表达情况，分析其与肿瘤病理生理特征的关系，以探讨自噬在肺癌发生发展及预后中的作用。

## 1 材料与方法

### 1.1 研究对象及临床资料

以2011年6月-2013年9月期间在天津医科大学总医院肺部肿瘤外科行肺癌切除术的患者，经术后病理组织学检查确诊为原发性NSCLC，且术前未经放疗、化疗和分子靶向治疗，共收集患者66例。病人的数据请见表 1，包括年龄、性别、吸烟史、病理类型、分化程度、淋巴结转移情况等。根据世界卫生组织（World Health Organization, WHO）第八版临床分期标准将患者的进行TNM分期。共包含男性46例，女性20例，年龄47岁-76岁，中位年龄61岁。其中腺癌45例，鳞癌21例。术后平均随访48.5个月（3个月-96.5个月），生存数据截止到2014年7月。这项研究通过了本院伦理委员会批准并取得患者的知情同意。

**1:** LC-3和P62在66例NSCLC中的表达情况和其临床特征分析 The expression of LC-3 and P62 in 66 lung cancer patients and their clinical characteristics

Factor	LC-3					P62		
		+	-	*P*		+	-	*P*
Total	66	27 (40.9%)	39 (59.1%)			43 (65.2%)	23 (34.8%)	
Histology								
SCC	21	12	9	0.013		5	16	< 0.001
AD	45	15	30			38	7	
Gender								
Male	46	20	26	0.432		30	16	0.38
Female	20	7	13			13	7	
Age (yr)								
≤61	19	6	13	0.889		10	9	0.076
> 61	47	21	26			33	14	
Smoking history								
Yes	30	11	19	0.409		22	8	0.112
No	36	16	20			21	15	
Clinical stage								
Ⅰ-Ⅱ	14	6	8	0.094		4	10	< 0.001
Ⅲ-Ⅳ	52	21	31			39	13	
Metastasis								
Yes	50	17	33	0.006		29	21	< 0.001
No	16	10	6			14	2	

### 1.2 免疫组织化学方法

66例肺癌组织标本均由天津医科大学总医院病理科常规固定、石蜡包埋，切片。4 μm-6 μm厚的石蜡病理切片，70 ℃烤片40 min，脱蜡，脱水后，经Tris/EDTA修复液（pH 8.0）进行抗原修复，正常羊血清RT封闭30 min，加入一抗4 ℃过夜，经生物素化的二抗37 ℃ 30 min，辣根酶标记的链霉素卵白素工作液37 ℃ 30 min，DAB显色液显色，苏木素复染。LC-3和P62抗体均购自Cell Signaling Technology，兔抗人单克隆抗体，经稀释液1:100稀释后使用。阳性对照由试剂公司提供，PBS代替一抗作为阴性对照。

### 1.3 免疫组织化学结果判定

每张切片随机选取10个高倍（400×）视野，每个视野计数100个细胞，根据细胞染色强度和阳性细胞比例评判实验结果。所有染色结果均由两位病理医生对患者的临床病理信息没有事先了解的情况下给出分数，然后在讨论后达成共识。对LC-3和p62染色强度和范围进行评分。根据染色强度分级为0（无染色）、1（轻度染色）、2（中度染色）、3（强染色）。根据染色的肿瘤细胞的百分比，评分如下：0（< 10%）、1（10%-49%），2（≥50%）。将两个得分相乘得到最终的结果，阳性（≥2）或阴性（< 2）。

### 1.4 统计学方法

采用SPSS 19.0统计软件包（IBM公司，美国）对所有数据进行统计学分析。运用卡方检验来分析LC-3和p62的表达与临床病理生理特征的关系。采用单变量和多变量*Cox*比例风险模型计算患者的危险比和95%CI。生存分析采用*Kaplan-Meier*法，以*P* < 0.05为差异具有统计学意义。

## 2 结果

### 2.1 LC-3与P62蛋白在NSCLC组织中的表达

如图 1所示，LC-3、P62阳性染色呈棕黄色或者黄褐色颗粒，在细胞质和细胞核中均有表达，阳性染色细胞排列呈巢状，周围被结蹄组织分割，另可见部分患者的细胞排列成腺腔样。在66例NSCLC患者中，27例LC-3染色阳性，阳性表达率为40.9%（27/66）; 43例P62染色阳性，P62的阳性表达率为65.2%（43/66）。

**1:**
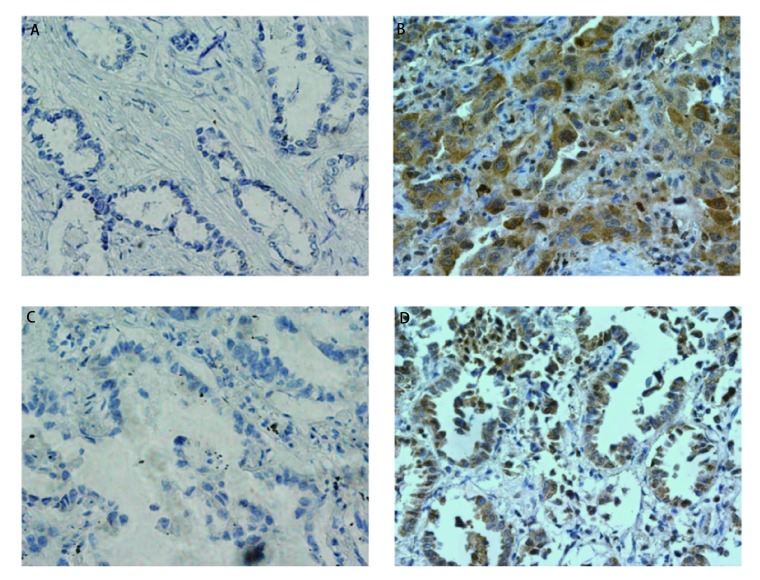
LC-3和P62免疫组化结果示意图。 The immunohistochemistry staining results of LC-3 and P62 in lung cancer.

### 2.2 LC-3与P62蛋白表达与NSCLC临床病理因素的关系

如表 1所示，我们进一步对LC-3和P62的表达情况与患者的临床病理生理特征进行分析，发现在不同组织学类型的患者中，LC-3和P62的表达情况存在明显差异：在鳞癌患者中LC-3阳性率为57.14%（12/21），明显高于其在腺癌患者中的阳性表达率（33.33%, 15/45）（*P* < 0.05）; 而P62却腺癌患者中的阳性表达率（84.44%, 38/45）明显高于其在鳞癌患者中的阳性表达率（23.81%, 5/21）（*P* < 0.05）。在Ⅲ期-Ⅳ期肺癌中P62的阳性表达率（75%, 39/52）明显高于其在Ⅰ期-Ⅱ期存肺癌中的表达（28.57%, 4/14）（*P* < 0.001）; 在不同转移状态的病人中，LC-3和P62的表达情况存在显著性差异：在有转移的患者中LC-3的阳性表达率仅为34%（17/50），明显低于其在无转移的患者中（62.5%, 10/16）（*P* < 0.001）; 而P62的阳性表达率在有转移的患者中为58%（29/50），虽然仍然明显低于其在无转移患者中的阳性表达率（87.5%, 14/16）（*P* < 0.001），但却明显高于LC-3在有转移患者中的阳性表达情况。

### 2.3 LC-3与P62蛋白在NSCLC中表达的相关性

如表 2所示，应用*Spearman*相关性分析显示：在66例NSCLC患者中，LC-3与P62均阳性者9例，LC-3与P62均阴性者5例，LC-3阳性P62阴性者18例，LC-3阴性P62阳性者34例，二者表达呈负相关（r_s_=-0.065, *P* < 0.001）。

**2:** LC-3和P62表达相关性分析 Correlation analysis of LC-3 and P62 expression

LC-3 expression	P62 expression		*P*
	Positive	Negative	
Positive	9	18	< 0.001
Negative	34	5	

### 2.4 LC-3和P62表达与其临床预后之间的关系

如图 2所示，应用*Kaplan-Meier*法对66例患者进行生存分析发现，LC-3蛋白阳性表达的患者预后明显好于其阴性表达的患者（39.1个月*vs* 16.3个月，*P*=0.0196）; 而P62蛋白表达水平却与患者的预后未见明显的相关性（阳性患者27.8个月*vs*阴性患者29.2个月，*P*=0.1629）。

**2:**
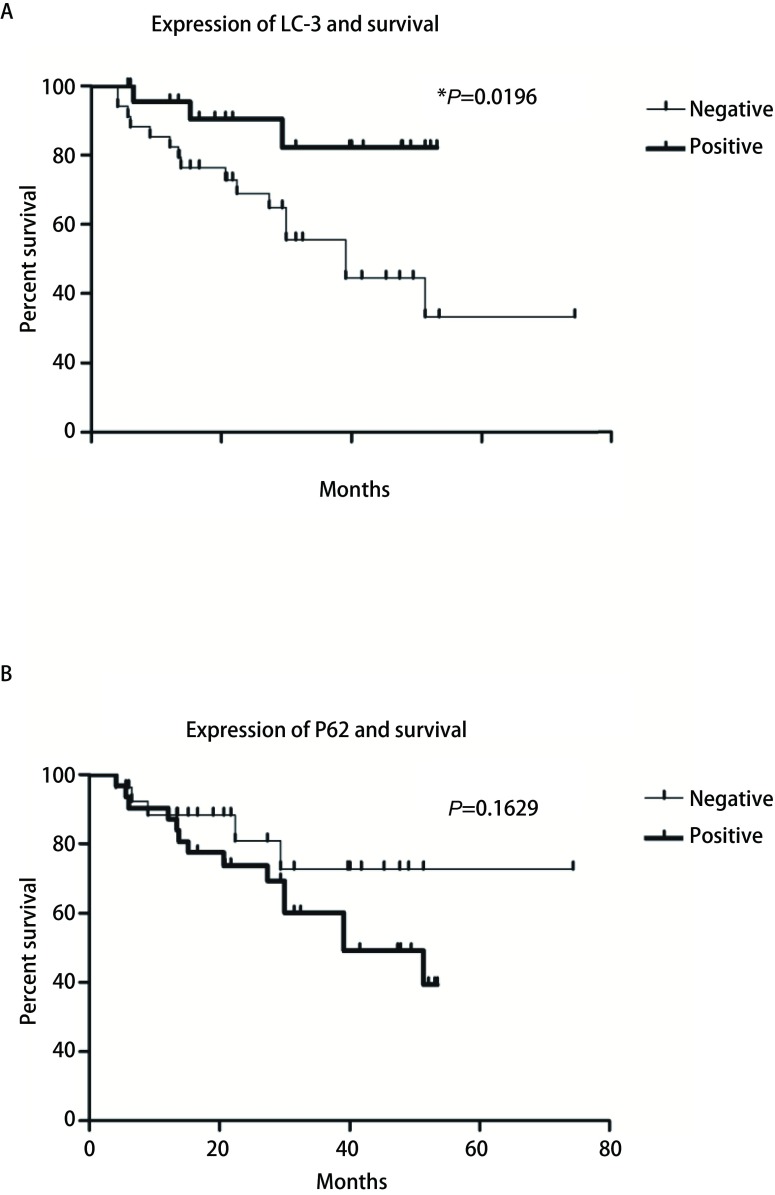
*Kaplan-Meier*生存曲线分析。 *Kaplan-Meier* survival analysis.

## 3 讨论

自噬是一种溶酶体依赖性的高效亚细胞降解途径，是广泛存在于真核细胞内的一种基本生命现象，它通过溶酶体将细胞多余的或无功能的物质降解，从而达到保护细胞内蛋白代谢平衡，调节细胞微环境稳态等目的^[8]^。许多研究表明，自噬对于肿瘤的发生发展可能起双重作用^[9-11]^。一方面，自噬通过维持肿瘤细胞内营养及微环境的稳态使得肿瘤细胞可以持续获得能量供给，有促进肿瘤生长的作用; 另一方面，自噬通过多种自噬标记性因子激活不同的细胞信号调节通路，使得肿瘤细胞发生程序性死亡，从而达到抑制肿瘤生长的作用。此外，自噬可导致肿瘤细胞对放疗、化疗和靶向治疗的耐受和治疗失败，从而导致肿瘤的进展、复发和转移。

LC-3是哺乳动物细胞中酵母ATG8基因的同源物，能定位于自噬体膜，参与自噬的形成，其表达产物有LC3 Ⅰ型和LC3 Ⅱ型两种形式。当自噬发生时，LC3Ⅰ型经泛素样加工修饰后与自噬体膜表面的磷脂酰乙醇胺结合，形成LC3 Ⅱ型。LC3 Ⅱ型蛋白结合并定位于胞内自噬体的膜上，其含量的多少与自噬泡的多少呈正比，可以间接反映细胞自噬水平的高低，是自噬的标记性分子^[12]^。P62是原癌基因*C-myc*编码，在细胞质中合成，随后表达于细胞核内的一种磷酸化蛋白质。P62可以与泛素化的蛋白质及自噬体膜上的LC-3 Ⅱ蛋白形成复合物，在自噬溶酶体内完成降解过程^[13]^。在自噬过程发生时，P62在细胞质内被不断的降解，P62表达降低; 当自噬流受阻时，P62蛋白在细胞质内聚集，表达量升高，因此，P62是一种可以反映自噬流活性的标记蛋白。

本研究结果显示，在NSCLC中，LC-3的阳性表达率为40.9%，在腺癌患者及有转移的患者中，LC-3的表达阳性率显著性低于鳞癌患者及无转移的患者，而LC-3较低表达的患者总体预后差，提示在NSCLC中，存在LC-3的表达异常并与患者的预后相关。Liu等的研究^[14]^结果显示，在NSCLC中LC-3的下调与患者的病理类型及远处转移情况有关，这与我们的研究结果一致。对于P62来说，在NSCLC中的阳性表达率为65.2%，在腺癌患者，TNM分期Ⅲ期、Ⅳ期及无远处转移的患者中P62的阳性表达率显著增高。进一步分析显示，P62与LC-3在NSCLC中的表达呈负相关（r_s_=-0.145, *P* < 0.01）。上述结果表明NSCLC组织中存在LC-3和P62表达异常，提示自噬可能介导NSCLC发生、发展过程。

综上所述，NSCLC中存在自噬相关蛋白LC-3和P62的表达异常，其表达情况与肿瘤的病理类型、临床分期及是否存在转移相关，提示自噬参与了NSCLC的发生发展过程，但其具体的作用机制仍需要进一步探讨。
